# Identification, characterization and comparative genomics of chimpanzee endogenous retroviruses

**DOI:** 10.1186/gb-2006-7-6-r51

**Published:** 2006-06-28

**Authors:** Nalini Polavarapu, Nathan J Bowen, John F McDonald

**Affiliations:** 1 School of Biology, Georgia Institute of Technology, Atlanta, Georgia 30332-0230, USA

## Abstract

**Background:**

Retrotransposons, the most abundant and widespread class of eukaryotic transposable elements, are believed to play a significant role in mutation and disease and to have contributed significantly to the evolution of genome structure and function. The recent sequencing of the chimpanzee genome is providing an unprecedented opportunity to study the functional significance of these elements in two closely related primate species and to better evaluate their role in primate evolution.

**Results:**

We report here that the chimpanzee genome contains at least 42 separate families of endogenous retroviruses, nine of which were not previously identified. All but two (CERV 1/PTERV1 and CERV 2) of the 42 families of chimpanzee endogenous retroviruses were found to have orthologs in humans. Molecular analysis (PCR and Southern hybridization) of CERV 2 elements demonstrates that this family is present in chimpanzee, bonobo, gorilla and old-world monkeys but absent in human, orangutan and new-world monkeys. A survey of endogenous retroviral positional variation between chimpanzees and humans determined that approximately 7% of all chimpanzee-human INDEL variation is associated with endogenous retroviral sequences.

**Conclusion:**

Nine families of chimpanzee endogenous retroviruses have been transpositionally active since chimpanzees and humans diverged from a common ancestor. Seven of these transpositionally active families have orthologs in humans, one of which has also been transpositionally active in humans since the human-chimpanzee divergence about six million years ago. Comparative analyses of orthologous regions of the human and chimpanzee genomes have revealed that a significant portion of INDEL variation between chimpanzees and humans is attributable to endogenous retroviruses and may be of evolutionary significance.

## Abstract 2

The identification and characterization of 42 families of chimpanzee endogenous retroviruses and a comparison to their human orthologs is described.

## Background

Retrotransposons are the most abundant and widespread class of eukaryotic transposable elements. For example, >30% of the mouse genome [1], >50% of the maize genome [2] and >60% of the human genome [3] are composed of retrotransposon sequences. This group of transposable elements is made up of short interspersed nuclear elements (SINEs), long interspersed nuclear elements (LINEs) and long terminal repeat (LTR) retrotransposons/endogenous retroviruses, all of which replicate via reverse transcription of an RNA intermediate [4]. The biological significance of retrotransposons ranges from their contribution to mutation (for example, [5]) and disease (for example, [6,7]) to their role in gene and genome evolution (for example, [8-10]).

The recent sequencing of the chimpanzee genome has provided an unprecedented opportunity to not only compare the full complement of retrotransposons in two closely related primate species but to gain insight into the role these elements may have played in human evolution. We have combined the use of an LTR retrotransposon search algorithm, LTR_STRUC [11], with a systematic series of iterative TBLASTN searches to identify the endogenous retroviruses present in the Ensembl chimpanzee database [12]. Since LTR_STRUC searches for LTR retrotransposons/endogenous retroviruses based on structure rather than homology, elements are often identified that go undetected in traditional BLAST searches (for example, [11]).

LTR_STRUC is designed specifically to find full-length LTR retrotransposons/endogenous retroviruses, that is, ones having two LTRs and a pair of target site duplications (TSDs) [11]. Thus, we complemented our search by using reverse transcriptase (RT) sequences from LTR_STRUC-identified elements as query sequences in an iterative series of TBLASTN searches. This allowed us to identify structurally aberrant elements not directly detected by LTR_STRUC. Finally, a series TBLASTN searches were carried out using, as query sequences, previously reported human RT sequences for which orthologues were not identified by our previous two searches.

## Results and discussion

### The chimpanzee genome contains at least 42 families of endogenous retroviruses

Using the procedure described above, we identified a total of 425 full-length chimpanzee endogenous retroviruses. This is certainly an underestimate of the number of endogenous retroviruses in the chimpanzee genome because we consciously excluded any sequences that could not be unambiguously identified as an endogenous retrovirus. The majority of these endogenous retroviruses (395/425 or 93%) were identified directly by LTR_STRUC or by homology to LTR_STRUC-identified elements.

**Figure 1:**
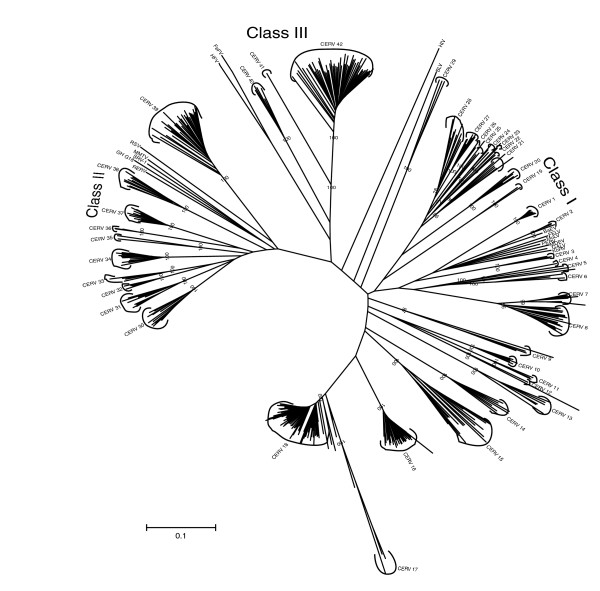
Unrooted RT based neighbor joining tree of three classes of chimpanzee endogenous retroviruses: class I, CERV1 to CERV29; class II, CERV30 to CERV 39; class III, CERV 40 to CERV 42. Bootstrap values are shown for each of the families. RT sequences from species other than chimpanzee, listed in Table 1, are included for comparison.

ClustalX [13] was used to build a multiple alignment of the RT domain of these 425 elements together with the RT domains of 16 previously described LTR retrotransposons/retroviruses representative of the three major classes of retroviral elements (Table 1). Phylogenetic analysis of the RT regions of the 425 full-length elements revealed the presence of at least 42 independent lineages of endogenous retroviruses in the chimpanzee genome that we here define as families (Figure 1). Non-autonomous endogenous retroviruses are elements that lack an RT open reading frame (ORF) and are required to utilize RT activity from autonomous, full-length endogenous retrovirus in order to replicate. Many of the chimpanzee endogenous retrovirus families contain truncated, non-autonomous as well as full-length elements.

Of the 42 families of chimpanzee endogenous retroviruses identified in this study, 40 were found to have orthologues in the human genome, including 9 that were identified in this study for the first time [14] (see Additional data file 1). Two previously identified chimpanzee endogenous retrovirus families do not have human orthologues (Table 2).

Consistent with the consensus nomenclature used for human endogenous retroviruses (HERV) [4], we here refer to the chimpanzee endogenous retroviral families by the acronym CERV (for chimp endogenous retrovirus). Distinct families are indicated by number (for example, CERV 1 to CERV 42). In the single instance where the CERV acronym refers to a previously named element/family, we include the pre-existing nomenclature as well (CERV 1/PTERV1). In those cases where a CERV family has an orthologue in humans, the name of the orthologous HERV family is given in parentheses (for example, CERV 3(HERVS71)).

### Endogenous retroviral families of the chimpanzee genome

LTR retrotransposons and retroviruses are grouped into three major classes [15]. Class I contains elements related to the gammaretroviruses (for example, Moloney murine leukemia virus (MuLV; accession no. AF033811), gibbon ape leukemia virus (GALV; accession no. M26927) and feline leukemia virus (FeLV; accession no. M18247)). Class II elements are related to betaretroviruses (for example, mouse mammary tumor virus (MMTV; accession no. NC_001503), rabbit endogenous retrovirus (RERV; accession no. AF480925)). Class III elements are distantly related to spumaviruses (for example, human foamy virus (HFV; accession no. Y07725), feline foamy virus (FeFV; accession no. AJ223851)). Of the 42 chimpanzee families identified in our study, 29 belong to class I, 10 to class II and 3 to class III (Figure 1).

While there is a precedence for classifying human endogenous retroviruses into families based on their tRNA primer-binding sites (for example, HERV K (lysine tRNA binding site)) [4], we find that such groupings do not accurately reflect the phylogenetic groupings of CERVs. For example, some members of the CERV 21 family have a proline tRNA binding site whereas other members of this same family utilize threonine tRNA as a primer. Conversely, phylogenetically divergent CERV families may share the same tRNA binding site (for example, members of the CERV 27 (HERV I) and CERV 30 (HERVK10) have lysine tRNA binding sites) (Table 2). Thus, primer binding sites appear to be an evolutionarily labile feature and thus not a reliable indicator of phylogenetic relationships among chimpanzee endogenous retroviruses. A similar conclusion has been drawn for LTR retrotransposons in *Caenorhabditis elegans *[16].

Full-length CERVs are typically between 7,000 and 10,000 base-pairs (bp) in length. Consistent with what has been reported for LTR retrotransposons/endogenous retroviruses in other species [17-19], CERV target site duplications (TSDs) range in size from 4 to 6 bp in length. With the exception of a few mutated copies, CERVs have the same canonical dinucleotides terminating the LTRs as have been reported for LTR retrotransposons/endogenous retroviruses in other species (TG/CA) [17-19]. CERV LTRs are typically 400 to 600 bp in length, although some LTRs are variant in size due to INDELs. For example, the LTRs of a member of the CERV 4 (HERV 3) family are 1,591 bp in length due to the insertion of an *Alu *element at some point in the evolutionary history of this lineage. The following is a more detailed characterization of the three classes of CERVs.

#### Class I: families 1 to 29

The CERV families 1 through 29 group with the class I retroviruses (Figure 1; Additional data file 2). The average size of full-length class I CERVs is 8,443 bp. These elements range in size from 2,268 to 13,135 bp in length. Much of this variation is due to INDELs associated with non-functional elements. The average size of LTRs associated with full-length class I CERV elements is 544 bp (range 195 to 1,591 bp). Class I CERV elements display considerable variation in their tRNA binding sites, even within families (Table 2). The most frequently used tRNA primer for class I CERV families (28%) is proline tRNA.

Because the LTRs of endogenous retroviruses are synthesized from a single template during reverse transcription, they are identical at the DNA sequence level upon integration [4]. Using the primate pseudogene nucleotide substitution rate of 0.16% divergence per million years [20,21], the relative integration time or age of CERV elements can be estimated from the level of sequence divergence existing between the element's 5' and 3' LTRs. The Jukes-Cantor model was used to correct for the presence of multiple mutations at the same site, back mutations and convergent substitutions [22]. Although caution must be taken when using LTR divergence to estimate the age of individual elements because of confounding processes such as recombination and conversion, (for example, [23,24]), the method is able to provide useful age estimates, at least to a first approximation (for example, [25]). Using this method, we estimate that the age of full-length class I CERV elements ranges from 0.8 to 82.9 million years (MY).

Full length elements representing at least three class I CERV families, CERV 1/PTERV1, CERV 2 and CERV 3 (HERVS71) have been recently transpositionally active as indicated by the presence of an unoccupied pre-integration site at the corresponding locus in humans. Inconsistent with this view is the fact that one of the chimpanzee-specific CERV 3 (HERVS71) insertions located on the Y chromosome displays an atypically high level of LTR-LTR sequence divergence (9%), indicative of it having inserted about 28 million years ago (MYA). However, the clear absence of this insert, both in the sequenced human genome (pre-integration site in tact) and in the genomes of several randomly sampled ethnically and geographically diverse humans (data not shown), indicates that this element most likely inserted after the chimpanzee-human divergence (about 6 MYA) and that the exceptionally high level of LTR-LTR sequence divergence is due to an inter-element recombination or conversion event [23,24]. All other class I CERV elements are much older and have not been reproductively active since well before chimpanzees and humans diverged from a common ancestor.

#### Class II: families 30 to 39

The CERV families 30 through 39 group with class II retroviruses (Figure 1; Additional data file 3). All Class II CERV families have orthologues in humans. The average size of full-length class II CERVs is 7,670 bp. This class of CERV elements range in size from 2,564 to 12,803 bp in length. As with class I elements, much of the size variation among class II elements is due to INDELs associated with non-functional elements. The average size of LTRs associated with full-length class II CERV elements is 544 bp (range 243 to 1,139 bp). Consistent with the fact that class II CERVs are orthologous to human HERV K elements, all but one family of class II CERV elements have lysine tRNA binding sites. The sole exception, CERV 39 (HERV K22), has a methionine tRNA binding site (Table 2). It has recently been proposed that HERV K22 be renamed HERV M to reflect its distinct primer binding site [26]. Unlike the other class II CERV elements, the CERV 39 (HERV K22) family clusters closely with the betaretrovirus (MMTV, SRV-1) (Figure 1; Additional data file 3).

**Figure 2:**
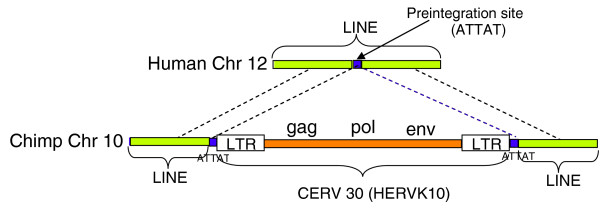
Insertion of a member of the CERV 30 (HERVK10) family in chimps. The insertion occurred in the LINE element present in chromosome 10 of the chimpanzee genome. The orthologous LINE element is present in chromosome 12 in humans. In chimpanzees target site duplications (ATTAT) are identified. A single copy of TSD (ATTAT, the pre-integration site) is found inside the LINE element in humans. The LTRs of the element are 99.4% identical.

The estimated age of full-length class II CERV elements ranges from 2 to 97 MY. A member of only one class II family, CERV 30 (HERV K10), has been transpositionally active since the divergence of chimps and humans from a common ancestor. The LTR sequence identity of one of the identified CERV 30 (HERVK10) elements is 99.4%, indicating that this element inserted into the chimpanzee genome about 2 MYA. We have verified that this CERV 30 (HERV K10) insertion is absent in humans (Figure 2). It has been previously reported [27,28] and we found in our INDEL analysis (see below) that at least 8 full-length copies of CERV 30 orthologue HERV K10, inserted into the human genome after the divergence of chimpanzees and humans from a common ancestor. In addition, two CERV 30 (HERV K10) insertion polymorphisms have been identified in human populations [29]. Thus, CERV 30 (HERV K10) family members and their human orthologues have been transpositionally active in both human and chimpanzee lineages since these species diverged from a common ancestor about 6 MYA.

CERV 36 (HERV K11D) is the second oldest family of class II CERV elements. We estimate that CERV 36 (HERV K11D) elements have not been transpositionally active for about 25 MY. We found that several members of the CERV 36 (HERV K11D) display the same deletion within the gag-pol regions of their genomes, suggesting that this deletion occurred prior to their transposition. Thus, this subfamily of CERV 36 (HERV K11D) elements comprised, at one time, non-autonomous elements and acquired essential replicative functions in *trans*.

#### Class III: families 40 to 42

The CERV families 40 (HERV S), 41 (HERV 16) and 42 (HERV L) group with class III retroviruses and are related to spumaviruses [4] (Figure 1; Additional data file 4). All class III CERV families have orthologues in humans. The average size of full-length class III CERVs is 6,758 bp. This class of CERV elements range in size from 2,980 to 13,271 bp in length. Again, much of this size variation is due to INDELs in this uniformly non-functional class of CERV elements. The average size of LTRs associated with full-length class III CERV elements is 446 bp (range 254 to 831 bp). CERV 40 elements have a serine tRNA binding site while CERV 42 elements have a leucine tRNA binding site (Table 2). Due to sequence ambiguities, we were unable to determine the tRNA binding site for CERV 41 elements (Table 2). Class III CERV elements are the oldest group of endogenous retroviruses in the chimpanzee genome. The estimated age of these elements ranges from 30 to 145 MY.

### Two CERV families have no human orthologues

#### CERV 1/PTERV1

With more than 100 members, CERV 1/PTERV1 is one of the most abundant families of endogenous retroviruses in the chimpanzee genome. CERV 1/PTERV1 elements range in size from 5 to 8.8 kb in length, are bordered by inverted terminal repeats (TG and CA) and are characterized by 4 bp TSDs (Table 2). The LTRs of the CERV 1/PTERV1 family of elements range from 379 to 414 bp in length. CERV 1/PTERV1 elements have a proline tRNA primer binding site (Table 2). LTR sequence identity among CERV 1/PTERV1 elements ranges from 97.1% to 99.7%.

**Figure 3:**
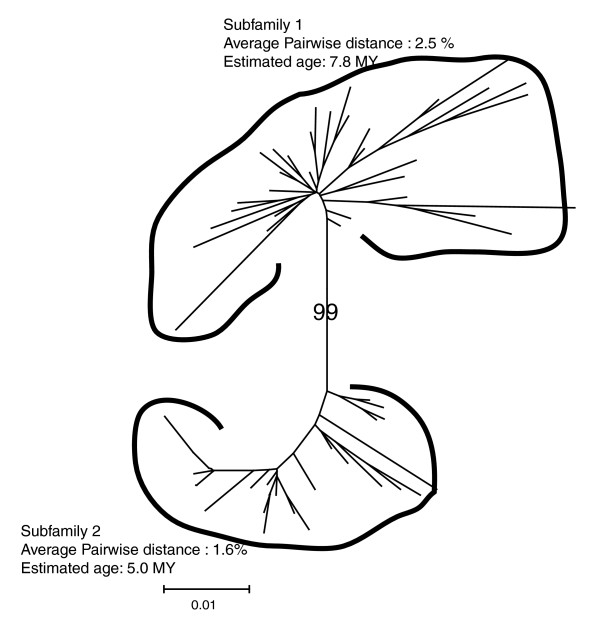
Phylogenetic tree of CERV 1/PTERV1 LTRs. Unrooted neighbor joining phylogenetic tree built from 5' and 3' LTRs from full-length CERV 1/PTERV1 elements. The average pairwise distances (corrected 'p' using Jukes-Cantor model) for each subfamily and the estimated ages are shown. Bootstrap values are shown.

Phylogenetic analysis of the LTRs from full-length elements of CERV 1/PTERV1 members indicated that this family of LTRs can be grouped into at least two subfamilies (bootstrap value of 99; Figure 3). The age of each subfamily was estimated by calculating the average of the pairwise distances between all sequences in a given subfamily. The estimated ages of the two subfamilies are 5 MY and 7.8 MY, respectively, suggesting that at least one subfamily was present in the lineage prior to the time chimpanzees and humans diverged from a common ancestor (about 6 MYA). This conclusion, however, is inconsistent with the fact that no CERV 1/PTERV1 orthologues were detected in the sequenced human genome. Moreover, we were able to detect pre-integration sites at those regions in the human genome orthologous to the CERV 1/PTERV1 insertion sites in chimpanzees, effectively eliminating the possibility that the elements were once present in humans but subsequently excised. Consistent with our findings, the results of a previously published Southern hybridization survey indicated that sequences orthologous to CERV 1/PTERV1 elements are present in the African great apes and old world monkeys but not in Asian apes or humans [30]. These results suggest that some members of the CERV 1/PTERV1 subfamily entered the chimpanzee genome after the split from humans through exogenous infections from closely related species and subsequently increased in copy number by retrotransposition. The unexpectedly high level of LTR-LTR divergence could be due to variation accumulated during the viral transfer [31] or possibly due to an inter-element recombination or conversion event subsequent to integration. Similar results were obtained when only the solo LTRs or both solo LTRs and LTRs from full-length elements were used in constructing the phylogenetic trees (Additional data files 5 and 6).

We found that a number of CERV 1/PTERV1 elements with high (>99%) LTR-LTR sequence identity have large (1 to 2 kb) deletions within the RT encoding region of their genomes. It is likely that these are non-autonomous elements that have inserted relatively recently by acquiring RT functions in *trans*, presumably from autonomous CERV 1/PTERV1 elements. Instances of recently inserted LTR retrotransposons/endogenous retroviruses lacking RT-encoding functions have previously been detected in the genomes of humans [32] and other species of both plants [18,33] and animals (for example, [16]).

#### CERV 2

This is the second family of chimpanzee endogenous retroviruses with no orthologue in the human genome. We identified ten solo LTRs and eight full-length copies of CERV 2 elements in the chimpanzee genome although, because of incomplete sequencing, we could identify the LTRs for only four of the eight full-length elements. CERV 2 elements are typically larger than CERV 1/PTERV1 elements, ranging in size from 8 to 10 kb in length. CERV 2 elements are bordered by inverted terminal repeats (TG and CA), have 4 bp TSDs (Table 2) and a proline tRNA primer binding site (Table 2). The LTRs of the CERV 2 family of elements range from 486 to 497 bp in length. Based on their LTR sequence identity (98.07% to 99.6%), we estimate that full-length CERV 2 elements were transpositionally active in the chimpanzee genome between 1.3 and 6.0 MYA. Thus, the majority of CERV 2 elements were biologically active after the divergence of chimpanzees and humans from a common ancestor.

**Figure 4:**
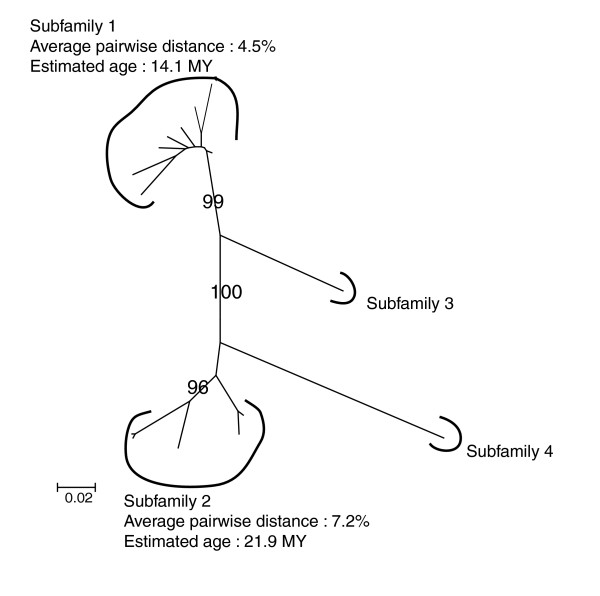
Phylogenetic tree of CERV 2 LTRs. Unrooted neighbor joining phylogenetic tree built from CERV 2 solo LTRs and 5' and 3' LTRs from full-length elements. The average pairwise distances (corrected 'p' using Jukes-Cantor model) for each subfamily and the estimated ages are shown. Bootstrap values are shown.

Phylogenetic analysis of solo LTRs and LTRs from full-length elements revealed that CERV 2 elements group into at least four subfamilies (bootstrap values >95; Figure 4). We estimated the ages of two of the more abundant subfamilies by calculating the average of the pairwise distances between all sequences in each subfamily. The estimated ages of the two subfamilies were 21.9 MY and 14.1 MY, respectively. As was the case for the CERV 1/PTERV1 family, these age estimates are inconsistent with the fact that no CERV 2 orthologues were detected in the sequenced human genome. Again, we were able to detect pre-integration sites at those regions in the human genome orthologous to the CERV 2 insertion sites in chimpanzees, effectively eliminating the possibility that the elements were once present in humans but subsequently excised.

**Figure 5:**
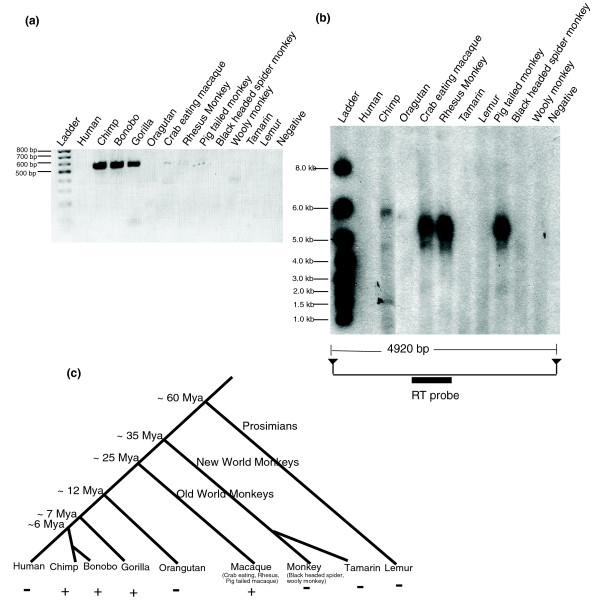
Distribution of CERV 2 elements among primates. Species surveyed include human (*Homo sapiens*), chimpanzee (*Pan troglodytes*), bonobo (*Pan paniscus*), gorilla (*Gorilla gorilla*), orangutan (*Pongo pygmaeus*), crab eating monkey (*Macaca fascicularis*), rhesus monkey (*Macaca mulatto*), pig tailed monkey (*Macaca nemestrina*), black headed spider monkey (*Ateles geoffroyi*), wooly monkey (*Lagothrix lagotricha*), red-chested mustached tamari (*Saguinus labiatus*), and ring-tailed lemur (*Lemur catta*). **(a) **PCR was conducted using primers designed in the RT region of chimpanzee CERV 2 element. The PCR results indicate that the CERV 2 element is present in chimpanzee, bonobo, gorilla and absent in other primates. **(b) **Southern hybridization was carried out on the DNA of the primates with negative PCR results using a probe designed in the RT region. The results indicate that CERV 2 like elements are present in chimpanzee, crab eating macaque, rhesus monkey and pig tailed monkey. Though the same amount of DNA was loaded in all lanes, the strength of hybridization is higher in old world monkeys than in chimpanzees, suggesting a higher copy number of CERV 2 elements in old world monkeys than in chimpanzees. Below the figure, a restriction map (chimpanzee sequence from chromosome 5 position 53871447.. 53880194 (NCBI build 1 version 1)) is presented in relation to the hybridization probe, *Hin*dIII (triangles). **(c) **The results from the combined PCR and Southern analyses demonstrate a patchy distribution of CERV 2 elements among primates.

We assessed the distribution of CERV 2 elements in primates by PCR using primers complementary to sequences in the conserved RT region. The results indicate that CERV 2 elements are present in chimpanzee, bonobo and gorilla but absent in human, orangutan, old world monkeys, new world monkeys and prosimians (Figure 5a). Southern hybridization experiments were carried out on DNA from species that gave negative PCR results to eliminate the possibility that the PCR primer binding sites have diverged in distantly related species within the CERV 2 RT and gag regions complementary to the designed probes (Figure 5b). The combined PCR and Southern analysis indicate that CERV 2 like sequences are present in chimpanzee, bonobo, gorilla and old world monkeys but absent in human, orangutan, new world monkeys and prosimians (Figure 5c). This distribution of CERV 2 elements among primates is identical to the above described distribution of CERV 1/PTERV1 elements [30]. It is worth noting that although the probes used in Southern hybridization were designed from chimpanzee element sequence, the strength of hybridization is higher in old world monkeys than in chimpanzees (Figure 5b), suggesting a higher copy number of CERV 2 elements in old world monkeys than in chimpanzees.

### Endogenous retroviral positional variation between chimpanzees and humans

Comparative analyses of orthologous regions of the human and chimpanzee genomes has revealed a number of instances where relatively large spans of sequence present in one species are not present in the other [34,35]. It has been proposed that these gaps or INDELs may be of evolutionary significance (for example, [9]). To determine the proportion of these gaps (human gaps are sequences present in chimpanzees but absent in humans; chimpanzee gaps are sequences present in humans but absent in chimpanzees) involving endogenous retroviruses, we utilized the human gap and chimpanzee gap datasets available at the UCSC Genome Bioinformatics web site [36] that were generated by aligning the chimpanzee genome with the human genome build HG16 [37,38]. These datasets include gaps of sizes ranging from 80 bp to 12.0 kb. Gap sequences from the datasets >5,000 bp (1,330 sequences), the typical length of full-length LTR retrotransposons/retroviruses, were blasted against the NCBI non-redundant protein database [39] using BlastX [40]. BLAST was used to identify species-specific full-length endogenous retroviral insertions in humans and chimpanzees. A total of 41 chimpanzee gap sequences and 31 human gap sequences were found to have significant similarity (e < 0.01) with retroviral sequences.

The presence of an endogenous retroviral sequence in chimpanzees that is missing at an orthologous genomic position in humans can be due to a novel insertion in chimpanzees or deletion of the element in humans. Similarly, the presence of an endogenous retroviral sequence in humans that is missing at an orthologous genomic position in chimpanzees can be due to novel insertion in humans or due to deletion of the element in chimpanzees. Because endogenous retroviruses do not precisely excise from insertion sites [4], it is possible to distinguish between these two possibilities. If a region in humans orthologous to the position of an endogenous retroviral insertion in chimpanzees contains a remnant of endogenous retroviral sequence (for example, fragmented element or solo LTR), we score the gap as a deletion in humans. If the orthologous region contains no remnant of the endogenous retrovirus but the pre-integration genomic sequence can be clearly identified, we score the gap as an insertion in chimpanzees. The same rules apply for the analogous dataset of the endogenous retroviral sequences present in humans but absent in chimpanzees.

**Figure 6:**
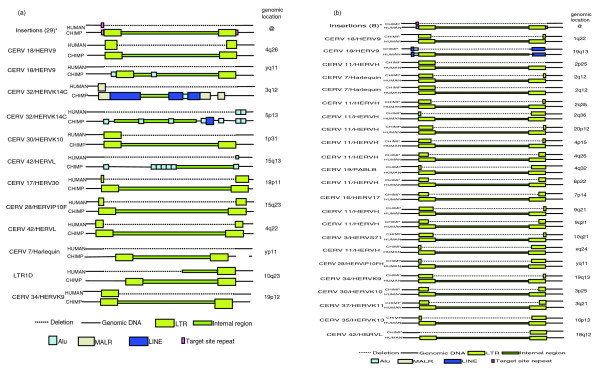
Structure of endogenous retroviral INDEL sequences (>5,000 bp) in humans and chimpanzees. **(a) **Characteristics of the remnant endogenous retroviral sequences (solo LTRs and/or fragmented elements) in humans. The asterisk indicates 29 chimpanzee specific endogenous retroviral insertions of which: 25 belong to the CERV 1/PTERV1 family (at genomic locations 5p12, 1q25, 12p13, 10q11, 3q11, 14q23, 2p11, 12p11, 4q13, 5q11, 3p22, 11p12, 13q12, 6q25, 7p14, 16p13, 7p21, 3q12, 12q12, 4p12, 5p14, 10q21, 7p14, 7p11, 5q12; 2 belong to the CERV 2 family (at genomic locations 3p24, 19q13); 1 belongs to the CERV 3 (HERVS71) family (at genomic location yp11]; and 1 belongs to the CERV 30 (HERVK10) family (at genomic location 12p13) (Table 3). **(b) **Characteristics of the remnant endogenous retroviral sequences (solo LTRs and/or fragmented elements) in chimps. The asterisk indicates 8 human specific endogenous retroviral insertions from the CERV 30 (HERVK10) family at genomic locations 22q11, 3q13, 3q27, 5q33, 6q14, 10q24, 11q22, 12q14 (Table 4).

Of the 41 instances where an endogenous retroviral sequence is present in chimpanzees but lacking in humans, 29 were due to novel insertions in chimpanzees while 12 were deletions in humans (Tables 3 and 4; Figure 6a). Of the 31 instances where an endogenous retrovirus is present in humans but absent in chimpanzees, we found that 8 were due to novel insertions in humans while 23 were deletions in chimpanzees (Table 4; Figure 6b). Of the 29 novel insertions in chimpanzees, 25 belong to the CERV 1/PTERV1 family, 2 to the CERV 2 family, 1 to the CERV 3 (HERVS7 1) family and 1 to the CERV 30 (HERVK10) family whereas all the 8 novel insertions in humans belong to the CERV 30 (HERVK10) family (Tables 3 and 4). Thus, four families of endogenous retroviruses have been transpositionally active in the chimpanzee lineage, resulting in full-length insertions, since chimpanzees and humans diverged from a common ancestor while only one of these families (CERV 30 (HERVK10)) has been active in humans (Tables 3 and 4). However, the family that is active in both humans and chimpanzees (CERV 30 (HERVK10)) generated eight novel full-length insertions in humans as opposed to only one novel insertion in chimpanzees since they diverged from the common ancestor (Tables 3 and 4).

Since solo LTRs and fragmented endogenous retroviral copies are typically ten to a hundred times more abundant than full-length elements in humans [14,41], we extended our survey to determine the extent to which INDEL variation between humans and chimpanzees is associated with solo LTRs and/or fragmented endogenous retroviral sequences. We again utilized datasets (human gaps and chimp gaps) available at the UCSC Genome Bioinformatics web site [36]. We used 'Repeat Masker' (AF Smit and P Green, unpublished data) to identify all interspersed repeats, that is, all transposable elements present in the datasets, and to subsequently extract endogenous retroviral homologous sequences.

Gap sequences were divided into two types: 'Mosaic type' gap sequences are defined as those composed of more than one category of interspersed repeats (for example, endogenous retrovirus inserted within a LINE element); and 'Single type' gap sequences are defined as those composed of only sequences homologous to endogenous retroviruses. Single type gap sequences were further divided into two categories: category 1 comprises those gap sequences composed entirely of an endogenous retroviral sequence; and category 2 comprises those gap sequences composed of endogenous retrovirus and non-interspersed repeat sequences. The above categorizations are useful in distinguishing gaps due to deletions in one species from the gaps due to insertions in the other species. Instances of mosaic type and single type category 2 gaps are deletions in that species while the gaps that belong to single type category 1 are either deletions in that species or insertions in the other species. Because endogenous retroviruses do not excise precisely [4] from the insertion sites, these later gaps can be further characterized as the result of insertions or deletions.

We found a total of 18,395 human gap sequences of which 9,855 (53.57%) contained interspersed repeats. Chimpanzees had a total of 27,728 gap sequences of which 15,652 (56.44%) contained interspersed repeats. A total of 1,495 human gap sequences contained endogenous retroviral sequences (592 mosaic type and 903 single type (640 category 1 + 263 category 2); Table 5). Five hundred and ninety two mosaic types and 263 single type category 2 were deletions in humans. Of the 640 single type gaps belonging to category 1, 152 are new insertions in chimpanzees while the remaining 488 are deletions in humans. Of the 152 chimpanzee insertions, 97 involved the two chimpanzee specific families while the remaining involved CERV families with human orthologues (Table 5).

A total of 1,608 chimpanzee gap sequences contained endogenous retroviral sequences (758 mosaic type and 850 single type (557 category 1+ 293 category 2). As stated above, 758 mosaic types and 293 single type category 2 are deletions in chimpanzees. Of the 557 that belonged to single type category 1, 79 are new insertions in humans while the remaining 478 are deletions in chimpanzees (Table 5).

Consistent with the copy number of CERV 30 (HERVK10) species-specific full-length insertions (Table 4), the insertions of solo LTRs from this family were higher in humans (78) than in chimpanzees (43) since they diverged from the common ancestor (Table 5). Apart from CERV 30 (HERVK10) insertions in both the genomes, five other endogenous retroviral families continued to be active in chimpanzees, resulting in solo LTR or fragmented insertions while only one new insertion from MER31B occurred in humans since they diverged from the common ancestor (Table 5).

## Conclusion

Once considered parasitic sequences of little or no adaptive significance [42,43], transposable elements are today generally recognized as significant contributors to human regulatory (for example, [44]) and structural (for example, [45]) gene evolution. The recent sequencing of the chimpanzee genome is providing a unique opportunity to conduct comparative genomic analyses of primate transposable elements.

Retrotransposons are the most abundant class of transposable elements. For example, retrotransposons comprise at least 60% of the human genome [3] and results presented here and elsewhere [34] suggest that the number of endogenous retroviruses in chimpanzees may be higher than in humans. In this paper, we present the results of the first systematic search for endogenous retroviruses in the chimpanzee genome. We have identified 425 full-length endogenous retroviruses in the chimpanzee genome that can be grouped into 42 independent lineages or families (Figure 1). All but two families of chimpanzee endogenous retroviruses were found to have orthologues in humans (Table 2). In contrast, we have found that all known families of human endogenous retroviruses have orthologues in chimpanzees. The two CERV families without orthologues in the human genome display a patchy distribution among primates (Figure 5) and our data suggest that at least some members of both families have been transpositionally active in the chimpanzee lineage after the divergence of chimpanzees and humans from a common ancestor.

We estimate that chimpanzee endogenous retroviruses range in age from about 0.8 to 145 MY. Nine families of chimpanzee endogenous retroviruses have been transpositionally active in chimpanzees while two families of human endogenous retroviruses have been transpositionally active in humans since they diverged from a common ancestor (Table 5). Thus, while some families of endogenous retroviruses have not been transpositionally active within the primate lineage, others have and continued to be active since chimpanzees and humans diverged from a common ancestor.

It has been estimated that 3.5% of the sequence differences between chimpanzees and humans is due to INDELs [34,35] and that this INDEL variation may be of particular evolutionary significance [9]. We have determined that approximately 7% of all chimpanzee-human INDEL variation is attributable to the presence or absence of endogenous retroviral sequences. The potential biological/evolutionary significance of this variation is currently under investigation.

Emerging evidence indicates that retrotransposons have played a significant role in gene and genome evolution (for example, [8-10]). The identification, characterization and comparative genomics of chimpanzee endogenous retroviruses presented in this report should not only help contribute to our understanding of the functional significance of these elements in chimpanzees but to a better appreciation of the role of endogenous retroviruses in primate evolution.

## Materials and methods

### Initial dataset scanning

The 2.73 GB chimpanzee genomic sequence [12] obtained from the Ensembl database was scanned for the presence of endogenous retroviruses using a structure based program, LTR_STRUC (LTR retrotransposon structure program) [11]. LTR_STRUC scans the genomic sequence for the presence of similar regions of length typical for LTRs (LTR pairs) and within the expected size of a full-length LTR retrotransposon/endogenous retrovirus. If the putative LTRs are found, the program then searches for additional retrotransposon features, such as primer binding sites (PBSs), poly-purine tracts (PPTs), target site repeats (TSRs), and assigns a reliability score to the hit based on the presence or absence of each of these features. A total of 2,056 hits were reported as the putative endogenous retroviruses in the chimpanzee genomic sequence, of which only 97 encoded RT.

### Sequence analysis for identifying the RT coding sequence

The 97 putative elements for which the presence of RT sequence was reported by LTR_STRUC were subjected to sequence analysis to identify the RT coding region. Briefly, sequence analysis involves aligning the amino acid sequence of the three reading frames reported by the search algorithm (the strand encoding RT protein is determined based on the presence of PBSs and PPTs) with previously annotated retroviral proteins using ClustalX [13] followed by manually checking of the three ORFs for the RT conserved motifs previously described [46,47]. From this sequence analysis we were able to identify RT conserved motifs for 25 hits.

### Identification of additional elements

The 25 RT sequences obtained from sequence analysis were augmented by conducting exhaustive sequence similarity searches using these sequences as queries against the 2.73 GB chimpanzee genomic sequence [12] using the TBLASTN program [40,48] to obtain an extensive set of endogenous retroviruses in the sequenced genome. Around 2,000 RT sequences were obtained by automatically parsing the TBLASTN search results for the hits above a threshold of 70% identity and covering a length of one-third of the query sequence using a perl script. After removing duplicates obtained during automatic parsing, we were left with 1,088 RT sequences.

### Identification of full length elements

The 1,088 RT sequences identified in the TBLASTN searches were checked for the presence of LTRs on either side of the RT as a criterion for full-length elements. This was done by examining the DNA sequences 7,000 bp on either side of the RT sequence, aligning them against each other using the program BLAST2SEQ and manually checking the hits for the presence of canonical dinucleotides, target site repeats and other LTR characteristic features. LTRs were identified for 395 of the 1,088 elements that had RT sequences. Thirty elements from previously reported human RT sequences for which orthologues were not identified in the above searches were added to our dataset, resulting in a total of 425 elements.

### Multiple sequence alignments and phylogenetic analysis

A multiple alignment was constructed from the DNA sequences of the RT region of 425 full-length elements together with representative members from the three classes of vertebrate retroviruses/LTR retrotransposons (Table 1) [4] using the program ClustalX [13]. We chose to use DNA sequence in making the multiple alignment and building the phylogenetic tree rather than amino acid sequence because of the presence of numerous frame shift mutations and stop codons in the elements. The multiple alignment was manually adjusted in the MEGA alignment browser [49]. A neighbor joining tree was generated from the alignment using MEGA2 with p-distance and pairwise deletions as parameters and bootstrap values were obtained from 1,000 replicates.

### Grouping the elements into families

The full-length elements were grouped into families based on the bootstrap values generated in the phylogenetic tree. Phylogenetically well supported clusters with high boot strap values were used to group the elements into families (Figure 1). The most recent element that is still intact is used as the representative element for each family (Table 2).

### Identification of the primer binding site

A 100 bp region downstream of the 5' LTR of full-length elements was searched against the chimp tRNA database downloaded from [50,51] using the program FASTA [52]. The 3' end of tRNA that matched with the reverse complement of the sequence over a stretch of 14 to 22 bp was assigned as a tRNA primer of the element (Table 2).

### Evolutionary analysis of CERV 1/PTERV1 and CERV 2 LTR sequences

Mulitple alignment was generated from the LTRs for each family using ClustalX [13]. A neighbor joining tree was generated from the alignment using MEGA3 [49] with Jukes-Cantor model [22] and pairwise deletions as parameters and bootstrap values were obtained from 1,000 replicates. The age of each subfamily was estimated by calculating the average of pairwise distances between all sequences in that subfamily and using the primate pseudogene nucleotide substitution rate of 0.16% divergence per million years [20,21].

### Molecular analysis: PCR and Southern hybridization

Primate DNA samples were purchased form Coriell cell repository (catalog no. PRP00001 and PRP00003) (Coriell institute for medical research, Camden, NJ, USA).

#### Polymerase chain reaction

Primers were designed in the conserved RT, gag, LTR and env regions of the CERV 2 element using the program PRIMER3 [53]. PCR amplification conditions were as follows: initial denaturation for 4.5 minutes at 94°C, 30 cycles of 30 s denaturation at 94°C, 30 s annealing at 57°C, 40 s elongation at 72°C and a final 1-cycle extension of 7 minutes at 72°C. The PCR products were then visualized on 1% (w/v) agarose gel.

#### Southern hybridization

Primate DNA was restriction enzyme digested, transferred to a nylon membrane and hybridized as described previously [54]. Nested PCR amplified products in RT and gag regions of CERV 2 elements were radioactively labeled and used as probes for hybridization. The same amount of DNA was loaded in all the lanes, with DNA samples in the order: human (*Homo sapiens*), chimpanzee (*Pan troglodytes*), orangutan (*Pongo pygmaeus*), crab eating monkey (*Macaca fascicularis*), rhesus monkey (*Macaca mulatto*), red-chested mustached tamari (*Saguinus labiatus*), ring-tailed lemur (*Lemur catta*), pig tailed monkey (*Macaca nemestrina*), black headed spider monkey (*Ateles geoffroyi*), wooly monkey (*Lagothrix lagotricha*).

## Additional data files

The following additional data are available with the online version of this paper. Additional data file 1 contains a description of the nine CERV families for which human orthologues were not identified previously. Additional data file 2 is a RT-based neighbor-joining tree for class I chimpanzee endogenous retroviruses. The distances (corrected 'p' using Jukes-Cantor model) appear next to each of the branches. RT sequences from species other than chimp, listed in Table 1, are included for comparison. The outgroup is class III element HFV (human foamy virus; see Table 1 and Additional data file 4). Additional data file 3 is a RT-based neighbor-joining tree for class II chimpanzee endogenous retroviruses. The distances (corrected 'p' using Jukes-Cantor model) appear next to each of the branches. RT sequences from species other than chimp, listed in Table 1, are included for comparison. The outgroup is class I element from CERV 1/PTERV1 family (see Table 2 and Additional data file 2). Additional data file 4 is a RT-based neighbor-joining tree for class III chimpanzee endogenous retroviruses. The distances (corrected 'p' using Jukes-Cantor model) appear next to each of the branches. RT sequences from species other than chimp, listed in Table 1, are included for comparison. The outgroup is class I element BaEV (baboon endogenous retrovirus; see Table 1 and Additional data file 2). Additional data file 5 is an unrooted neighbour joining phylogenetic tree built from solo LTRs and 5' and 3' LTRs of full-length elements of the CERV1/PTERV1 family. Bootstrap values are shown on the tree. The average pairwise distances (corrected 'p' using Jukes-Cantor model) for each subfamily and the estimated ages are shown. Additional data file 6 is an unrooted neighbour joining phylogenetic tree built from solo LTRs of the CERV 1/PTERV1 family. Bootstrap values are shown in the tree. The average pairwise distances (corrected 'p' using Jukes-Cantor model) for each subfamily and the estimated ages are shown.

## Supplementary Material

Additional data file 1 A description of the nine CERV families for which human orthologues were not identified previously Click here for file

Additional data file 2 The distances (corrected 'p' using Jukes-Cantor model) appear next to each of the branches. RT sequences from species other than chimp, listed in Table 1, are included for comparison. The outgroup is class III element HFV (human foamy virus; see Table 1 and Additional data file 4). Click here for file

Additional data file 3 The distances (corrected 'p' using Jukes-Cantor model) appear next to each of the branches. RT sequences from species other than chimp, listed in Table 1, are included for comparison. The outgroup is class I element from CERV 1/PTERV1 family (see Table 2 and Additional data file 2). Click here for file

Additional data file 4 The distances (corrected 'p' using Jukes-Cantor model) appear next to each of the branches. RT sequences from species other than chimp, listed in Table 1, are included for comparison. The outgroup is class I element BaEV (baboon endogenous retrovirus; see Table 1 and Additional data file 2). Click here for file

Additional data file 5 Bootstrap values are shown on the tree. The average pairwise distances (corrected 'p' using Jukes-Cantor model) for each subfamily and the estimated ages are shown. Click here for file

Additional data file 6 Bootstrap values are shown in the tree. The average pairwise distances (corrected 'p' using Jukes-Cantor model) for each subfamily and the estimated ages are shown. Click here for file

## Figures and Tables

**Table 1:** Previously characterized RT sequences from a variety of species used for comparison in phylogenies

Name	Name of retrovirus	Accession number
RERV	Rabbit endogenous retrovirus	AF480925
GH-G18	Golden hamster intracisternal A-particle H18	GNHYIH
SRV-1	Simian SRV-1 type D retrovirus	M11841
MMTV	Mouse mammary tumor virus	NC_001503
RSV	Rous sarcoma virus	AF052428
HFV	Human foamy virus	Y07725
FeFV	Feline foamy virus	AJ223851
HIV-1	Human immunodeficiency virus 1	K03454
BLV	Bovine leukemia virus	K02120
BaEV	Baboon endogenous virus	X05470
FELV	Feline leukemia virus	M18247
MuLV	Moloney murine leukemia virus	AF033811
PERV	Porcine endogenous retrovirus	AF038601
MDEV	Mus dunni endogenous virus	AF053745
GALV	Gibbon ape leukemia virus	M26927
KoRV	Koala type C endogenous virus	AF151794

Also see Figure 1 and Additional data files 2-4

**Table 2:** Representative sequences from each family of chimpanzee endogenous retroviruses

Family name: chimp family (orthologous human family)	tRNA primer	Location on chromosome (chromosome no: position)	5' and 3' LTR % identity	Length of 5'/3' LTRs (bp)	Target site repeats	Dinucleotides	Element length (bp)
CERV 1/PTERV1	Pro	8:62466629..62474817	99.7	409/409	GTAT/GTAT	TG/CA	8,189
CERV 2	Pro	1:53871490..53880190	98.8	497/486	GTGA/GTGA	TG/CA	8,338
CERV 3 (HERVS71)	Thr	7:45002408..45013133	90	528/524	AGGC/AGGC	TG/CA	10,726
CERV 4 (HERV3)	Arg	6:65506183..65515842	91.7	643/592	TATA/TATA	TG/CA	9,660
CERV 5 (HERV15)	Thr	20:22151622..22161290	90	495/500	TTTT/TTTT	TG/CC	9,669
CERV 6 (HERV 1*)	Thr	8:43017710..43027603	92	511/512	CCAC/CCAC	TG/CA	9,697
CERV 7 (Harlequin)	Glu	14:49265903..49274634	90	477/470	ATAAAT/ATAAAT	TG/CA	8,745
CERV 8 (HERVE)	Glu	4:29336385..29342031	92.3	354/355	AACA/AACA	TG/CA	5,647
CERV 9 (HERV 2*)	His	1:74420643..74424449	74.5	318/315	CTTTT/CTTTT	TG/CA	3,807
CERV 10 (HERVH48)	Phe	1:162967211..16293841	86	404/401	ATTCT/ATTCT	TG/CA	6,640
CERV 11 (HERV-H)	His	13:88231927..88241255	91	367/367	TGTTA/TGTTA	TG/CA	9,329
CERV 12 (HERVFH19)	ND	1:9084130..9093376	88	412/421	ND	ND	9,236
CERV 13 (PRIMA4)	Arg	X:81351532..81361722	86	627/617	CCTC/CCTC	TG/CA	10,191
CERV 14 (HERV 5*)	Leu, Arg	3:58020412..58027601	83	420/430	CACT/CACT	TG/CA	7,198
CERV 15 (HERV-P)	Pro, Val	6:89330483..89339138	87	634/625	ATACC/ATACT	TG/CA	8,500
CERV 16 (HERV-17)	Gln, Arg	4:55955342..55963662	89	775/760	CCTT/CCTT	TG/CA	8,329
CERV 17 (HERV30)	Leu, Arg	5:121436599..121446300	89	698/700	AAAG/AAAG	TG/CA	9,702
CERV 18 (HERV 9)	Arg, Lys, Pro	5:39234784..39242752	87	423/449	GGAG/GGAG	TG/CA	7,966
CERV 19 (PABL_B)	Arg, Leu	7:75174020..75182429	83	671/667	AGAG/AGAG	TG/CA	8,410
CERV 20 (HERVP71A)	Pro	X:121795847..121803454	85	464/458	TTTTC/TTTTC	TG/CA	7,608
CERV 21 (HERV 4*)	Thr, Pro	2:14653540..14661848	91	361/363	ATGA/ATGA	TG/CA	8,321
CERV 22 (HERV 6*)	Thr	16:84359558..84368114	86	434/435	ND	AG/CT	8,557
CERV 23 (HERV 7*)	Pro	17:39042670..39052505	88	582/575	AGAC/AGAC	TG/CA	9,836
CERV 24 (HERV 10*)	Pro	17:27621756..27630879	87	429/431	TAAT/TAAT	TG/CA	9,132
CERV 25 (HERV 11*)	Pro	1:32316520..32325874	84	613/621	GCAAA/GCAAA	TG/CA	9,480
CERV 26 (HERV 12*)	ND	2:171938873..171948150	93	509/506	AATT/ACTT	TG/CA	9,279
CERV 27 (HERVI)	Lys	5:122623439..122630725	89.9	497/506	CAGT/CAGT	CG/CA	7,287
CERV 28 (HERVIP10F)	Ala	23:41095392..41106316	95.6	494/496	TACT/TACT	TG/CA	10,925
CERV 29 (HERVG25)	ND	6:151527383..151531731	84	220/226	ND	ND	4,349
CERV 30 (HERVK10)	Lys	10:4757815..4766975	99.4	961/958	ATTAT/ATTAT	TG/CA	9,161
CERV 31 (HERVK14)	Lys	9:74609341..74617943	92	623/618	CAATG/CAATG	TG/CA	8,603
CERV 32 (HERVK14C)	Lys	12:84993042..85001650	92	583/583	ND	TG/CA	8,609
CERV 33 (HERVK(C4))	Lys	1:134530572..134538281	94	541/547	ATTAAG/ATTAAG	TG/CA	7,710
CERV 34 (HERVK9)	Lys	X:58520547..58526579	93.4	510/508	GCCTAG/GCCTAG	TG/CA	6,033
CERV 35 (HERVK13)	ND	19:41852728..41865530	83	812/818	ND	ND	12,803
CERV 36 (HERVK11D)	Lys	5:123901088..123908580	91	865/874	ATAAAT/ATAAAT	TG/TA	7,493
CERV 37 (HERVK11)	Lys	2:81402412..81411338	95.7	1,079/1,079	ATAAAA/ATAAAA	TG/CA	8,927
CERV 38 (HERVK3)	Lys	5:26871940..26880204	95.2	428/431	GGTAAA/GGTGAA	TG/CA	8,265
CERV 39 (HERVK22)	Met	5:103484321..103492150	85	477/497	GTTCTT/GTTCTT	TG/CA	7,830
CERV 40 (HERV S)	Ser	1:147219818..147226527	85	325/329	CCATC/CCATC	TG/CA	6,710
CERV 41 (HERV16)	ND	X:103812023..103815002	ND	ND	ND	ND	2,980
CERV 42 (HERVL)	Leu	3:83740925..83746481	82	445/458	ATAAT/ATAAT	TG/CA	5,547

*Families submitted to Repbase. ND, not determined.

**Table 3:** Endogenous retrovirus INDEL sequences (>5000 bp) present in chimpanzees but absent in humans

Human gaps	Gap sequence
25 (I)	CERV 1/PTERV1
2 (I)	CERV 2
2 (D)	CERV 18 (HERV9)
2 (D)	CERV 32 (HERVK14C)
2 (1 I + 1 D)	CERV 30 (HERVK10)
2 (D)	CERV 42 (HERVL)
1 (D)	CERV 17 (HERV30)
1 (I)	CERV 3 (HERVS71)
1 (D)	CERV 28 (HERVIP10F)
1 (D)	CERV 7 (Harlequin)
1 (D)	LTR1D
1 (D)	CERV 34 (HERVK9)

I, insertion in chimps; D, deletion in humans

**Table 4:** Endogenous retrovirus INDEL sequences (>5000 bp) present in humans but absent in chimpanzees

Chimp gaps	Gap sequence
9 (8 I +1 D)	CERV 30 (HERVK10)
10 (D)	CERV 11 (HERVH)
2 (D)	CERV 18 (HERV9)
1(D)	CERV 16 (HERV17)
1 (D)	CERV 34 (HERVK9)
1 (D)	CERV 37 (HERVK11)
1 (D)	CERV 35 (HERVK13)
1 (D)	CERV 3 (HERVS71)
1 (D)	CERV 42 (HERVL)
2 (D)	CERV 7 (Harlequin)
1 (D)	CERV 28 (HERVIP10F)
1 (D)	CERV 19 (PABL_B)

I, insertion in humans; D, deletion in chimps.

**Table 5:** Endogenous retrovirus INDELs (80 bp to 12.0 kb) in humans and chimpanzees

	Human	Chimpanzee
Total Gaps	18,395	27,728
Gaps with interspersed repeats	9,855	15,652
Gaps containing endogenous retrovirus sequences	1,495	1,608
Mosaic gaps	592	758
Single gaps	903	850
Category 2 gaps	263	293
Category 1 gaps	640	557
Deletions	488	478
Insertions	79	152
CERV 1/PTERV1	-	85 (25 full ln + 60 solo LTRs)
CERV 2	-	12 (2 full ln + 10 solo LTRs)
CERV 30 (HERVK10)	78 (8 full ln + 70 solo LTRs)	43 (1 full ln + 42 solo LTRs)
CERV 3 (HERVS71)	-	1 (1 full ln)
CERV 11 (HERVH/LTR7)	-	1*
CERV 37 (HERVK11/MER11C)	-	1*
CERV 34 (HERVK9/MER9)	-	1*
CERV 18 (HERV9)	-	7*
CERV 35 (HERVK13/LTR13)	-	1*
MER31B	1*	-

*Solo LTRs and/or fragmented copies. ln, length.
