# Supplementary material for: Identification, characterization and comparative genomics of chimpanzee endogenous retroviruses
Source: Genome Biol. 2006 Jun 28;7(6):R51. doi: 10.1186/gb-2006-7-6-r51 (PMC1779541; doi:10.1186/gb-2006-7-6-r51)
Supplement: Additional data file 1 — A description of the nine CERV families for which human orthologues were not identified previously [file gb-2006-7-6-r51-S1.doc]

#### **CERV families with previously unrecognized human orthologues**

Nine novel Class I CERV families with previously unidentified orthologues in the human genome are characterized in this paper for the first time (Table 1).

**CERV 6:** We identified ~21 full-length elements, fragmented copies and solo LTRs of CERV 6 in the chimp genome. CERV 6 elements range in size from 9.6 – 10.1 kb in length, are bordered by inverted terminal repeats (TG and CA) and have 4 – 5 bp TSD (Table 1). CERV 6 elements have a threonine tRNA primer binding site (Table 1). The LTRs of the CERV 6 family of elements range from 511 to 649 bp in length. Based on their LTR sequence identity (92.16 % to 96.53 %), we estimate that these CERV 6 elements inserted into the primate lineage between 10.87 –25.86 MYA, i.e., well before the divergence of chimps and humans from a common ancestor.

**CERV 9:**  CERV 9 elements are among the oldest LTR retrotransposons in the chimp genome. Approximately 8 full-length elements, fragmented copies and solo LTRs of CERV 9 were found in the chimp genome. CERV 9 elements range in size from 3.8 – 4.2 kb in length, are bordered by inverted terminal repeats (TG and CA) and have 5 bp TSD (Table 1). CERV 9 elements have a histidine tRNA primer binding site (Table 1). The LTRs of the CERV 9 family of elements range from 195 to 318 bp in length. Based on their LTR sequence identity (68.63 % to 88.36 %), we estimate that these CERV 9 elements inserted into the primate lineage between 36.38 –98.05 MYA.

**CERV 14:** Approximately 170 full-length elements, fragmented copies and solo LTRs of CERV 14 were found in the chimp genome. CERV 14 elements range in size from 7.1 – 9.8 kb in length, are bordered by inverted terminal repeats (TG and CA) and have 4 bp TSD (Table 1). CERV 14 elements have either a leucine or arginine tRNA primer binding site (Table 1). The LTRs of CERV 14 family of elements range from 305 to 459 bp in length. Based on their LTR sequence identity (80.5 % to 86.42 %), we estimate that these CERV 14 elements inserted into the primate lineage between 42.44 –60.93 MYA.

**CERV 21:** Approximately 27 full-length elements, fragmented copies and solo LTRs of CERV 21 were identified in the chimp genome. CERV 21 elements range in size from 8.0 – 10.8 kb in length, are bordered by inverted terminal repeats (TG and CA) and have 4 bp TSD (Table 1). CERV 21 elements have either a threonine or a proline tRNA primer binding site (Table 1). The LTRs of CERV 21 family of elements range from 364 to 637 bp in length. Based on their LTR sequence identity (87.8 % to 91.2 %), we estimate that these CERV 21 elements inserted into the primate lineage between 27.5 – 37.9 MYA.

**CERV 22:** Approximately 40 full-length elements, fragmented copies and solo LTRs of CERV 22 were found in the chimp genome. CERV 22 elements range in size from 5.9 – 8.5 kb in length, are bordered by inverted terminal repeats (AG and CT). Because of the accumulation of substitutions at the borders of two elements in this family, it was not possible to accurately determine TSDs for this family (Table 1). CERV 22 elements have a threonine tRNA primer binding site (Table 1). The LTRs of CERV 22 elements range from 256 to 431 bp in length. Based on their LTR sequence identity (86.6 % to 90.6 %), we estimate that these CERV 21 elements inserted into the primate lineage between 29.5 – 41.9 MYA.

**CERV 23:** We identified approximately 36 full-length elements, fragmented copies and solo LTRs of CERV 23 in the chimp genome. CERV 23 elements range in size from 9.2 – 9.8 kb in length, are bordered by inverted terminal repeats (TG and CA) and have 4 bp TSD (Table 1). CERV 23 elements have a proline tRNA primer binding site (Table 1). The LTRs of CERV 23 elements range from 575 to 681 bp in length. Based on their LTR sequence identity (84.7 % to 87.6 %), we estimate that these CERV 23 elements inserted into the primate lineage between 38.6 – 47.8 MYA.

**CERV 24:** Approximately 65 full-length elements, fragmented copies and solo LTRs of CERV 24 were found in the chimp genome. CERV 24 elements range in size from 8.6 – 9.1 kb in length, are bordered by inverted terminal repeats (TG and CA) and have 4 bp TSD (Table 1). CERV 24 elements have a proline tRNA primer binding site (Table 1). The LTRs of the CERV 24 family of elements range from 427 to 437 bp in length. Based on their LTR sequence identity (88.2 % to 89.1 %), we estimate that these CERV 24 elements inserted into the primate lineage between 34.0 – 37.0 MYA.

**CERV 25:** Approximately 67 full-length elements, fragmented copies and solo LTRs of CERV 25 were found in the chimp genome. CERV 25 elements range in size from 9.5 – 9.9 kb in length, are bordered by inverted terminal repeats (TG and CA) and have 5 bp TSD (Table 1). CERV 25 elements have a proline tRNA primer binding site (Table 1). The LTRs of the CERV 25 family of elements range from 549 to 617 bp in length. Based on their LTR sequence identity (90.3 % to 94.3 %), we estimate that these CERV 25 elements inserted into the primate lineage between 17.91 - 30.34 MYA.

**CERV 26:** Approximately 33 full-length elements, fragmented copies and solo LTRs of CERV 26 were found in the chimp genome. CERV 26 elements range in size from 9.3 – 10.9 kb in length, are bordered by inverted terminal repeats (TG and CA) and 4 bp TSD (Table 1). Because of sequencing ambiguities, it was not possible to determine the tRNA binding site for CERV 26 elements (Table 1). The LTRs of the CERV 26 family of elements range from 494 to 509 bp in length. Based on their LTR sequence identity (93.4 % to 93.9 %), we estimate that these CERV 26 elements inserted into the primate lineage between 19.1 – 20.7 MYA.
